# Characterization of Haptoglobin Isotype in Milk of Mastitis-Affected Cows

**DOI:** 10.3390/vetsci3040029

**Published:** 2016-10-13

**Authors:** Indu Upadhyaya, Jacob Thanislass, Anitha Veerapandyan, Sharanabasav Badami, Prabhakar X. Antony

**Affiliations:** 1 Department of Poultry Science, University of Arkansas, Fayetteville, AR 72701, USA; 2 Department of Veterinary Biochemistry, Rajiv Gandhi Institute of Veterinary Education and Research, Puducherry 605009, India; jthanis@rediffmail.com (J.T.); dranivet85@gmail.com (A.V.); drbaddy4u@gmail.com (S.B.); 3 Department of Veterinary Microbiology, Rajiv Gandhi Institute of Veterinary Education and Research, Puducherry 605009, India; pxantony@gmail.com

**Keywords:** haptoglobin, mastitis, subclinical, diagnosis, local origin, biomarker

## Abstract

Haptoglobin is a major acute phase protein in bovines and reportedly increases in serum and milk whey during mastitis, highlighting its potential as a diagnostic biomarker. Since haptoglobin is known to undergo tissue specific glycosylation resulting in different isoforms, this study was undertaken to characterize the isoforms of haptoglobin. Milk whey fraction and serum obtained from animals with or without clinical mastitis in Puducherry, India, were subjected to SDS-PAGE followed by western blot and immuno-detection of haptoglobin protein. All subunits (β, α1 and α2) of haptoglobin protein were detected in serum sample obtained from clinical cases. However, only the β-subunit was detected in milk whey fraction obtained from the respective animals. Similar results were observed with milk whey fractions from subclinical cases indicating difference in isoform of haptoglobin detected in milk whey from serum. This was further supported by RT-PCR (Reverse Transcription Polymerase Chain Reaction) analysis of haptoglobin gene (Hp) confirming the tissue specific origin of haptoglobin.

## 1. Introduction

Mastitis is the most prevalent infectious disease among dairy cattle, and is responsible for huge financial losses to the dairy industry worldwide [1,2]. Subclinical mastitis does not express any clinical sign of the disease, but the growth, reproductive performance and milk yield of the animal are affected, which results in major economic losses to cattle production. Hence, in a well-managed dairy herd, in addition to clinical mastitis, subclinical mastitis is important and should be efficiently detected [3]. The presence of clinical mastitis is easy to assess, whereas the diagnosis of the subclinical form can be more difficult and requires special laboratory assays [4]. In recent years, much effort has been invested in the search for biomarkers to diagnose mastitis, one such group of markers being the acute phase proteins (APP). APPs are a group of serum proteins that undergo substantial quantitative changes in response to infection, inflammation, or trauma [5]. APPs, as alternative biomarkers of mastitis, may increase in concentration in the absence of macroscopic changes in the milk, or may precede the onset of clinical signs, thereby aiding in rapid diagnosis of subclinical infection [6]. More recently, APPs such as haptoglobin (Hp) and serum amyloid A (SAA), measured in blood and, more importantly, in milk, have been highlighted as potential diagnostic markers for bovine mastitis [7,8,9]. However, its role as a marker in subclinical mastitis has been minimally studied.

Hp is a major APP in cattle and its serum concentration can increase up to 1000 fold during infection, when compared to a moderate (5–10-fold increase) response observed in humans and swine [10,11]. Hp and somatic cell count (SCC) are significantly elevated in bovine milk following intra mammary administration of endotoxin or bacteria [12]. Previous studies conducted to validate the efficacy of various APPs as diagnostic markers have indicated that measuring Hp and SAA in milk is more accurate than measuring other APPs and also more reliable than serum analysis of the above parameters for the diagnosis of subclinical mastitis [6]. The studies conducted thus far investigating the expression of mRNA for Hp have suggested that mammary tissue could be a source of APPs in bovine milk [13]. The mammary epithelial cells represent an additional extra-hepatic source of Hp and function as a possible source of Hp in milk [14]. Further, it has been stated that neutrophils and epithelial cells may play an essential role in elevating milk Hp [12]. There is also evidence that Hp is expressed in various extra hepatic tissues like lung, kidney, skin and heart [15,16]. Moreover, there is a plausibility of formation of tissue specific isoforms of Hp further supporting the mammary origin of Hp [17]. Hence, the present study was undertaken to further characterize the Hp isoforms detected in milk and specifically focus on the role of Hp as a biomarker of subclinical mastitis.

## 2. Materials and Methods

### 2.1. Sample Preparation and Bacteriological Examination

Milk samples were collected from healthy and mastitis-affected cattle from four farms across Puducherry, India, and subjected to California Mastitis Test (CMT) [18] to predict the somatic cell count (SCC) of milk. The CMT is a screening test for subclinical mastitis that can be used easily at cowside. The use of the CMT to identify infected quarters has been extensively validated in cows that do not demonstrate clinical signs of mastitis and are in early lactation [19]. The CMT was performed as per Dingwell et al. 2003 [18] to predict the somatic cell count of milk. Equal volume of CMT reagent was added to the milk sample and agitated. When a mixture remained unchanged on agitation, it was presumed to have an SCC of ~100,000 and was categorized as normal (N). A stringy mass on agitation was categorized as M^+^ with an SCC OF ~900,000, whereas a slight gel formation seen on agitation was categorized as M^++^ with an SCC OF ~2.7 million. An almost solid gel formation seen on agitation was categorized as M^+++^ with a presumed SCC of ~8.1 million. The bacteriological examination was conducted in our lab previously [20]. The milk samples were streaked onto Mueller Hinton Agar and incubated at 37 °C for 24–48 h. A minimum of 5 colonies of same type was recorded as causative agent from which individual colonies were subjected to gram staining. Identification of the gram positive cocci arranged in clusters were identified and the corresponding milk samples were confirmed as *Staphylococcus aureus* by Polymerized Chain Reaction (PCR) of *nuc* gene. Blood samples from both clinically normal animals and those affected with mastitis were collected in sterile vials without any anticoagulant. The serum was collected and stored at −80 °C for further Hp protein detection with western blot.

### 2.2. Isolation of Total RNA from Milk

The samples were pelleted by centrifugation at 1000× *g* for 20 min at 4 °C. The fat layer was discarded and the supernatant containing the whey fraction was stored at −80 °C for western blot analysis. The pellet obtained was washed thrice with ice cold sterile Phosphate Buffered Saline (PBS, pH 7.2, HiMedia, India), and centrifuged at 1000× *g* for 15 min and the supernatant was discarded. After the final wash, the milk somatic cells were resuspended in PBS and total RNA was isolated as per the manufacturer’s protocol, using TRI reagent (Sigma-Aldrich Inc., St. Louis, MO, USA).

### 2.3. RT-PCR (Reverse Transcription Polymerase Chain Reaction) and Western Blot for Haptoglobin Characterization

Concentration of serum and milk whey fractions were determined by spectrophotometry and subjected to SDS–PAGE (SE 260, General Electric (GE) healthcare, Chennai, India). The gel containing 12% polyacrylamide (Cat# 92560, Sigma-Aldrich) was used with a top stacking gel of 5% polyacrylamide. Approximately 100 μg of protein from serum and whey were loaded per well onto the gels and each tested sample was preheated at 95 °C for 15 min in a loading buffer (12 mM TrI-HCl, pH 6.8, 0.4% SDS (Cat# L3771, Sigma-Aldrich), 5% glycerol (Cat# G5516, Sigma-Aldrich), 0.02% bromphenol blue (Cat# B0126, Sigma-Aldrich) with 140 mM 2-mercaptoethanol (Cat# M3148, Sigma-Aldrich). The samples were run for about 45 min at 150 V and subjected to Silver staining. This was followed by western blot of the fractioned proteins onto a nitro-cellulose membrane (Sigma-Aldrich) in a immunoblot unit (TV100-EBK, SCIE-PLAS, Banglore, India), at 150 V for two hours. Hp protein was detected using rabbit polyclonal antibodies (Abcam, Cambridge, UK) against Hp with a 1:1000 dilution in assay buffer. The goat anti-rabbit Immunoglobulin G-horseradish peroxidase (IgG-HRP) conjugate (Bangalore Genei Pvt. Ltd., Bangalore, India) and 3,3′-diaminobenzidine (DAB, Cat# D12384, Bangalore Genei Pvt. Ltd.) were used for color development.

The total somatic milk cell RNA was subjected to formaldehyde agarose gel electrophoresis (2%) and the distinct bands corresponding to 28s and 18s rRNA were identified (Supplementary Materials Figure S1). Additionally, RNA at A260/A280 was tested for each sample and the samples with a ratio of 2.00 were selected for further processing. The select RNA samples (2 µg/µL) were subjected to a one step RT-PCR for mRNA amplification of Hp gene with published specific primers [13]. The actin gene [21] expression served as a positive control. The amplified products were detected by agarose gel (2%) electrophoresis and semi-quantified using Quantity One software (Bio-Rad, Chennai, India).

### 2.4. Statistical Analysis

Fifteen milk samples from each category of normal, M^+++^, M^++^, and M^+^ were selected for the study with corresponding serum samples. For each experiment (RT-PCR and Western Blot), a minimum of 6 biological replicates were used with three technical replicates. All experiments were repeated twice. For the RT-PCR, semi-quantification was done in Quantity one Software and analyzed using one-way anova.

## 3. Results and Discussion

Increased serum Hp concentration is indicative of inflammation [22], tissue injury [23] and is often accompanied by change in Hp glycosylation associated with disease development and progression [24]. Hp in its simplest form consists of two α- and two β-chains, connected by disulfide bridges. The chains originate from a common precursor protein, which is proteolytically cleaved during protein synthesis [25]. Bovine Hp consists of monomers of 16 to 23 kDa (α-chains) and 35 to 40 kDa (β-chains) and exists as a polymer in association with albumin with a molecular weight above 1000 kDa in cattle serum [10,26].

**Figure 1:**
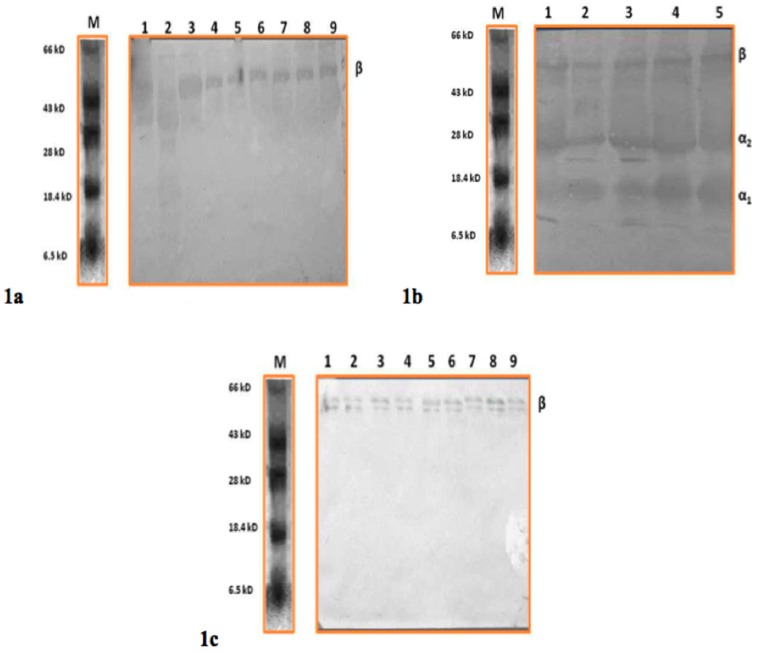
(**a**) Western Blot detection of Haptoglobin (Hp) in milk whey from clinical mastitis. Lane 1 to 9: Milk whey from clinical mastitis cases; Lane M: Molecular weight marker; (**b**) Western Blot detection of Hp in serum from clinical mastitis. Lane 1 to 5 Serum from clinical cases; Lane M: Molecular weight marker; (**c**) Western Blot detection of Hp in milk whey from subclinical mastitis. Lane 1 to 3: M^+^; Lane 4 to 6: M^++^; Lane 7 to 9: M^+++^; Lane M: Molecular weight marker.

This study aimed to identify the origin of milk Hp, in order to further establish the role of Hp as a diagnostic marker for mastitis. To ascertain the origin, western blot analysis of milk whey protein and serum was carried out for clinical mastitis (Figure 1a,b) and subclinical cases (Figure 1c) of Hp protein. In Figure 1a,c, the polyclonal antibody raised against Hp protein could detect only the β-subunit of Hp protein. However, the antibody could detect all the subunits of Hp protein (β, α1 and α2 corresponding to molecular sizes 35–40 kDa, 9 kDa and 16–23 kDa, respectively) in serum samples of clinical mastitis (Figure 1b) from the same animal. All sub units of Hp were observed on the blot when serum from mastitis cows was subjected to western blot (Figure 1b). This is consistent with previous studies, which have demonstrated the presence of α and β-chains in Hp of bovine granulocytes indicating four and five isotypes respectively [27]. However, in the milk whey protein, only β subunits were predominantly detected in clinical samples (Figure 1a) and in subclinical mastitis cases (Figure 1c).

Smeets and co-workers [28] demonstrated the presence of unique set of glycoforms of Hp in arteries when compared to Hp produced in liver attributing to a difference in post-translational modification. We detected two different isoforms of Hp from the serum and milk of the same animal. All the subunits of Hp protein (β, α1 and α2 corresponding to molecular sizes 35–40 kDa, 9 kDa and 16–23 kDa, respectively) were detected in serum samples of clinical mastitis. However, in the milk whey protein, only β subunits were predominantly detected in both clinical and subclinical samples. In addition, the mRNA expression in somatic cells also supports this finding. Hence we observed that the Hp measured in milk was potentially from the mammary tissue and not from the blood. Our results suggest the presence of isotypes of Hp protein in cattle and also the possibility of Hp detected in milk whey being different from that detected in serum. Appearance of Hp in milk has been considered to be either due to leakage of Hp protein from systemic circulation attributed to an altered vascular permeability or potential local synthesis of Hp [29]. Results of our study indicate that Hp found in milk may have originated from the mammary tissue of cattle.

**Figure 2:**
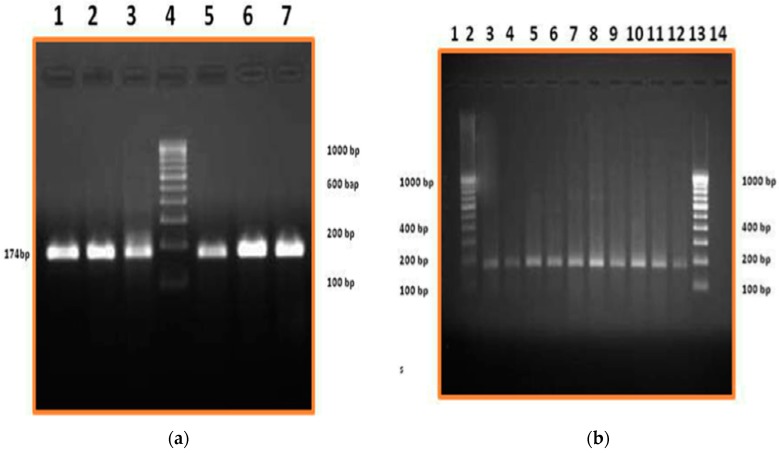
(**a**) Hp Gene Expression in clinical mastitis cases. Lane 1 to 3 and Lane 5 to 7: RT-PCR product from clinical mastitis cases; Lane 4: 100 bp DNA ladder; (**b**) Hp Gene Expression in subclinical mastitis cases. Lane 3 and 4: M^+^; Lane 5 and 6: M^++^; Lane 7 to 12: M^+++^; Lane 2 and Lane 13: 100 bp DNA ladder.

In addition, the local (mammary) origin of milk Hp protein was further supported by mRNA expression of Hp gene in milk somatic cells as observed in Figure 2a,b. There was no expression of Hp mRNA or the protein (serum and milk) from normal (N) samples (Figure S2) as Hp is an APP expressed during infection. Hp mRNA (174 bp) was detected as early as M^+^ stage of subclinical mastitis and the expression increased with the degree of mastitis condition, highlighted in Figure 2b. In addition, semi quantification of Hp mRNA expression using Quantity One Software revealed a significant increase in Hp gene expression from subclinical state of M^+^ to clinical mastitis state (Figure S3).

Although the role of Hp in clinical mastitis has been investigated previously [30,31], the isotypes of Hp in subclinical mastitis infections have been minimally studied. Our work highlighted three different haptoglobin isoforms that could be potentially addressed as specific molecular features for diagnosis. The current study provides additional evidence substantiating the origin of milk Hp with regards to its isoforms supporting the suitability of milk Hp as a diagnostic marker of subclinical mastitis.

## Supplementary Materials

The following are available online at www.mdpi.com/2306-7381/3/4/29/s1, Figure S1: Expression of total RNA for milk samples: Different lanes correspond to milk samples extracted from animals with mastitis. RNA from Lane 1, 3 and 4 was considered for further processing whereas RNA from lane 2 and 5 was degraded. Additionally, the ratio of samples at A260/280 from Lane 1 and 3 was confirmed to be ~2.00 prior to subjecting them for RT-PCR, Figure S2: Western Blot detection of Hp in milk whey from normal and subclinical mastitis. Lane 1: normal (N); 2: M^+^, Lane 3 to 5: M^+++^, Figure S3: mRNA Expression of Hp gene using Quantity One software. In this figure, x-axis denotes the samples and y-axis denotes the brightness/trace intensity when compared to Normal (0/baseline). 1: Sub-Clinical mastitis case (M^+^); 2: Sub-Clinical mastitis case (M^++^); 3: Sub-Clinical mastitis case (M^+++^); 4: Clinical mastitis case. a—In comparison with M^+^; b—In Comparison with M^++^; c—In comparison with M^+++^. *** denotes *p* < 0.001.
