# Supplementary material for: Characterization of Haptoglobin Isotype in Milk of Mastitis-Affected Cows
Source: Vet Sci. 2016 Oct 13;3(4):29. doi: 10.3390/vetsci3040029 (PMC5606594; doi:10.3390/vetsci3040029)
Supplement: Supplementary file 1 [file vetsci-03-00029-s001.pdf]

Supplementary Materials: Characterization of Haptoglobin Isotype in Milk of Mastitis-Affected Cows

Indu Upadhyaya, Jacob Thanislass, Anitha Veerapandyan, Sharanabasav Badami and Prabhakar X. Antony

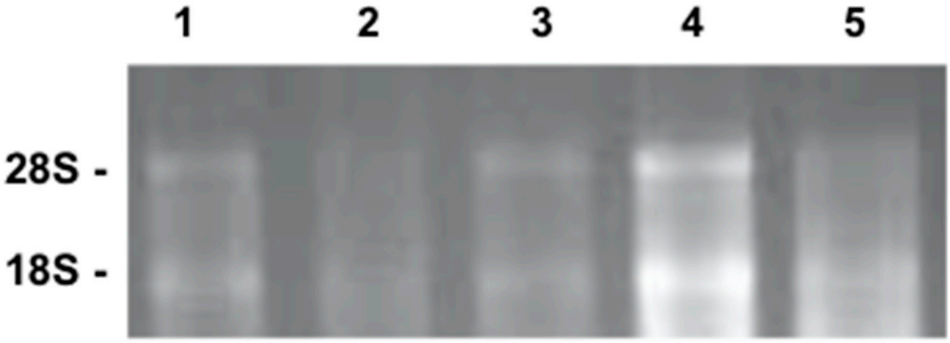

Figure S1. Expression of total RNA for milk samples: Different lanes correspond to milk samples extracted from animals with mastitis. RNA from Lane 1, 3 and 4 was considered for further processing whereas RNA from lane 2 and 5 was degraded. Additionally, the ratio of samples at A260/280 from Lane 1 and 3 was confirmed to be ~2.00 prior to subjecting them for RT-PCR.

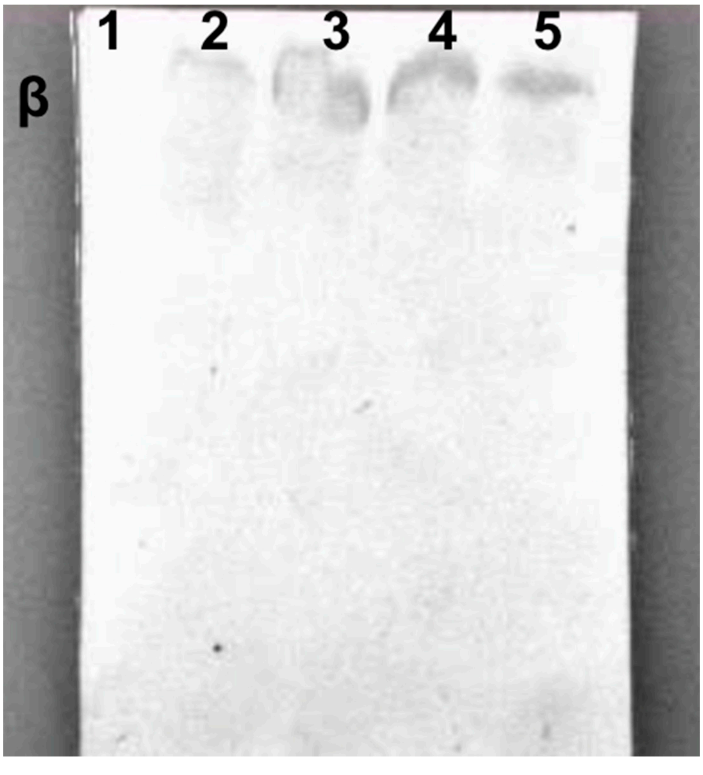

Figure S2. Western Blot detection of Hp in milk whey from normal and subclinical mastitis. Lane 1: normal (N); 2: M^{+}, Lane 3 to 5: M^{+++}.

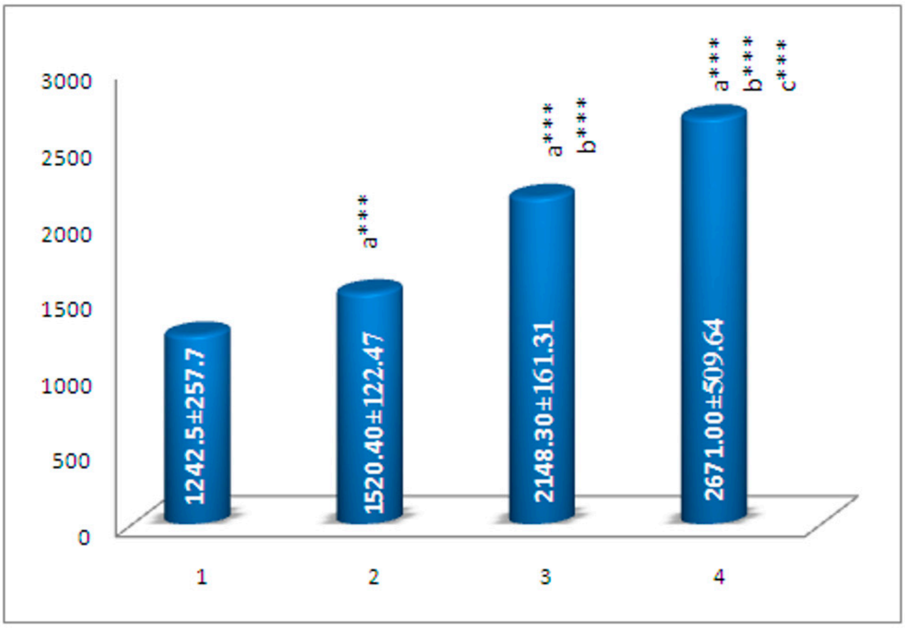

Figure S3. mRNA Expression of Hp gene using Quantity One software. In this figure, x-axis denotes the samples and y-axis denotes the brightness/trace intensity when compared to Normal (0/baseline). 1: Sub-Clinical mastitis case (M^{+}); 2: Sub-Clinical mastitis case (M^{++}); 3: Sub-Clinical mastitis case (M^{+++}); 4: Clinical mastitis case. a—In comparison with M^{+}; b—In Comparison with M^{++}; c—In comparison with M^{+++}. *** denotes $p < 0.001$.
